# IS THERE A DIFFERENCE BETWEEN RIGHT- VERSUS LEFT-SIDED COLON CANCERS? DOES SIDE MAKE ANY DIFFERENCE IN LONG-TERM FOLLOW-UP?

**DOI:** 10.1590/0102-672020190001e1479

**Published:** 2019-12-20

**Authors:** Leonardo Alfonso BUSTAMANTE-LOPEZ, Sergio Carlos NAHAS, Caio Sergio R. NAHAS, Rodrigo Ambar PINTO, Carlos Frederico S. MARQUES, Ivan CECCONELLO

**Affiliations:** 1 Hospital das Clínicas, School of Medicine, Gastroenterology, University of São Paulo, São Paulo, SP, Brazil

**Keywords:** Colonic neoplasms, Colorectal surgery, Laparoscopy, Neoplasias intestinais, Cirurgia colorretal, Laparoscopia

## Abstract

**Background:**

Since 1990 it was proposed that distal and proximal location of colon cancer might follow different biological, epidemiology, pathology and prognosis, probably due to embryologic different development of the two segments of the colon, which may represent two separate disease entities. These differences might have consequences for the treatment of patients with colorectal cancer.

**Aim:**

To compare the characteristics between patients with right and left colon cancer, with severity and tumor characteristic that influence in the survival of these patients.

**Method:**

Were evaluated the outcomes of surgical treatment of patients with colon cancer with data collected retrospectively from prospectively collected database.

**Results:**

The tumor’s side did not influence survival time of patients with colon cancer (p=0.112) in the regression model. Only the diseases stage leads to influence on survival time; patients with right colon cancer have more advanced staging (III or IV) and present a risk of death greater in 3.23 times.

**Conclusion:**

This analysis provides evidence that the prognosis of localized left-sided colon cancer is better compared to right-sided colon cancer. Also, the patients with right colon cancer have more advanced stage, mucinous tumor and are older.

## RESUMO

**Racional:**

Desde 1990, foi proposto que a localização distal e proximal do câncer de cólon pode seguir diferentes aspectos biológicos, epidemiológicos, patológicos e prognósticos. Essas diferenças podem ter consequências para o tratamento de pacientes com câncer colorretal.

**Objetivo:**

Comparar as características entre pacientes com câncer de cólon direito e esquerdo, com gravidade e características tumorais que influenciam na sobrevida desses pacientes.

**Método:**

Avaliação dos resultados do tratamento cirúrgico dos pacientes com câncer de cólon em longo prazo com dados coletados retrospectivamente.

**Resultados:**

O lado do tumor não influenciou o tempo de sobrevida (p=0,112) no modelo de regressão. Apenas o estágio da doença influencia no tempo de sobrevida. Os pacientes com câncer de cólon direito apresentam estadiamento mais avançado (III ou IV) e apresentam risco de morte 3,23 vezes maior.

**Conclusão:**

O prognóstico do câncer de cólon localizado no lado esquerdo é melhor comparado ao direito. Os pacientes com câncer de cólon direito têm estágio e idade mais avançados e tumor mucinoso.

## INTRODUCTION

Colorectal cancer (CRC) is the third most common cancer in the USA and the second cause leading to cancer deaths although intense screening programs have declined the incidence rate.

Since 1990 it was proposed that distal and proximal location of colon cancer might follow different biological, epidemiology, pathology and prognosis^2^ ^,^ ^5^ ^,^ ^9^. This it may well be to the different embryologic development of the two segments of the colon, which may represent two separate disease entities^3^. These differences might have consequences for the treatment of patients with colorectal cancer^33^.

Differences have been noted in the following characteristics: right-sided colon cancers are more likely to be exophytic, to be diploid, and to have mucinous histology and high microsatellite instability, whereas left-sided colon cancers are often infiltrating lesions, present with obstructive symptoms, and are more often aneuploid^5^ ^,^ ^15^ ^,^ ^23^ ^,^ ^35^.

The objective of this study was to compare the characteristics between patients with right and left colon cancer, with severity and tumor characteristic that influence in the survival of these patients in long-term clinical follow-up.

## METHODS

This study was approved by the institutional ethics committee and numbered as 090819.

For the elaboration we evaluated the outcomes of surgical treatment of patients with colon cancer treated in the Service of Colon and Rectal Surgery of the *Hospital das Clínicas*, School of Medicine of University of *São Paulo, SP, Brazil,* from 2002 - 2012. Data were collected retrospectively from prospectively collected database. Of 1219 patients treated, a total of 566 patients had colon cancer. Of these, 187 underwent right hemicolectomy and 173 left colectomy with curative intent, thus making the total number of patients included in this study. Patients with incomplete data, synchronous cancers, or with benign disease were previously excluded from the analysis.

The following factors were studied and compared between the right and left colon cancer: gender, age, tumor location, number of nodes removed in the specimen, lymph node status, T stage and presence of distant metastases. Also these factors were evaluated for the possibility of prognostic impact in 5-year survival.

The clinicopathologic and tumor characteristics were studied with the use of summary measures (mean, standard deviation, median, minimum and maximum) for quantitative variables and absolute and relative frequencies for qualitative variables.

### Statistical analysis

For the overall survival, mean survival times were estimated according to the characteristics of interest using the Kaplan-Meier function. It was not possible to estimate the median time because the number of deaths occurred was less than 50% and compared the overlaps between categories using log-rank tests. The Hazard Ratios (HR) was estimated with the respective 95% confidence intervals using the bivariate Cox regression. For the variables that influenced bivariate in the overall survival, it was verified the joint influence of the same ones in the survival with the use of the Cox multiple regression. Two models were created, one with separate staging in T, N and M and one with final staging of the patient.The characteristics of the patients according to the location of the colon tumor (right and left) were compared using Student’s t or Mann-Whitney tests for the quantitative variables and chi-square tests to verify the existence of association with the qualitative variables.A multiple Cox regression model was created with all patients, controlling the characteristics that presented significance in the survival of the patients of each group and with the variables that differed between the groups to verify if the side of the tumor influences the survival of the patient with colon cancer.The tests were performed with significance level of 5%.

## RESULTS

Of the 370 patients with colon cancer 187 were right and 171 left sided. Were compared the outcomes of the two groups of patients (Table 1). 

**TABLE 1:** Description of characteristics of the patients and the tumor side of the colon cancer and statistical tests

Variable	Colon cancer side		p
	Right (n: 187)	Left (n: 173)	
Age (years) Mean (SD) Median (min ; max)	65.05 (12.11) 66 (31;88)	60.82 (13.22) 63 (22 ; 87)	0.002*
In-hospital time (days) mean (SD) median (min.; max.)	16.64 (9.83) 14 (5; 60)	16.91 (9.01) 14 (5; 60)	0.294**
Gender, n (%) Female Male	105 (56.1) 82 (43.9)	85 (49.1) 88 (50.9)	0.183
T stage, n (%) T1 T2 T3 T4	9 (4.8) 14 (7.5) 140 (74.9) 24 (12.8)	15 (8.7) 19 (11.0) 111 (64.2) 28 (16.2)	0.467**
N stage, n (%) N0 N1	101 (54) 86 (46)	101 (58.4) 72 (41.6)	0.404
M, n (%) Negative Positive	180 (96.3) 7 (3.7)	167 (96.5) 6 (3.5)	0.889
Stage, n (%) I II III IV	9 (4.8) 79 (42.2) 92 (49.2) 7 (3.7)	25 (14.5) 78 (45.1) 64 (37.0) 6 (3.5)	0.004**
Lymph node, n (%) Lessthan 12 12 or more	24 (12.8) 163 (87.2)	32 (18.5) 141 (81.5)	0.139
Histologicaltype, n (%) Non mucinousMucinous	158 (84.5) 29 (15.5)	163 (94.2) 10 (5.8)	0.003
Tumor differentiation, n (%) Well Poorly	166 (88.8) 21 (11.2)	163 (94.2) 10 (5.8)	0.066

Chi-square test; * Student´s t-test; ** Mann-Whitney´s test

Table 1 shows that patients with right colon cancer were significantly older (p=0,002), with more advanced final stage (p=0,004) and with more cases of mucinous histological type (p=0,003) when compared with left colon cancer. Others factors like gender, in-hospital time, lymph nodes and tumor differentiation didn’t had any significant difference. The 5-years survival was 78% for the patients with left and 70.9% for the right colon cancer.

Table 2 shows that the tumor’s side did not influence survival time of patients with colon cancer (p=0.112) in the regression model. Only the diseases stage leads to influence on survival time; patients with right colon cancer have more advanced staging (III or IV) and present a risk of death 3.23 times higher.

**TABLE 2:** Multiple Cox regression model

Variable	HR	95% CI		p
		Lower	Upper	
Age (years)	1.01	0.99	1.02	0.581
Gender (Male)	1.02	0.66	1.55	0.944
Stage (III or IV)	3.23	2.01	5.17	<0.001
Sidecolon CA (Left)	0.70	0.45	1.09	0.112

## DISCUSSION

Our study shows in the univariable methods that right colon cancer had a significant difference in the age of the diagnosis, more advance stage at the moment of the surgery and more patients with mucinous type of tumor. At the multivariable analysis only advanced stage was more common in right colon cancer patients.

 Currently, data regarding the prognosis of right-sided vs. left-sided colon cancer are conflicting; however, most studies revealed a poorer survival in right-sided primary tumor location

 Saltzstein et al.^30^, concluded that increasing age was associated with a shift of anatomic site of origin of CRC from the left to right side of the colon. This is in accordance with the results of the present paper, where the median age at diagnosis of right colon cancer was 65 years vs. 60 in left colon cancer (Table 1).

In the present study we only included patients with elective surgery. It has previously been shown that emergency resection is associated with an increased morbidity and postoperative mortality compared with elective resection^18^.

We found a significant difference in the advance stage of patients with right colon cancer, without discrepancies in tumor, lymph node and metastases stage alone. According to Hemminki et al.^13^, patients with RCC had more advanced stages at diagnosis than patients with LCC, and Snaebjornsson et al.^34^ found that the more advanced stages of RCC were due to tumor (T) and lymph node (N) stages, but not to metastases (M stage).

RCC was associated with a larger number of harvested lymph nodes and a larger amount of positive lymph nodes among these than LCC^2^ ^,^ ^8^ ^,^ ^21^ ^,^ ^40^ ^,^ ^41^. The number of resected and positive lymph nodes is a quality parameter, as lymph node metastases are of imperative significance to prognosis and treatment^28^ ^,^ ^29^. Our media of LN in our patients was 23 without any difference when compare left and right colon cancer.

 In our previous publications we studied the division of the right and left colon cancer^24^ ^,^ ^25^. In a more recent paper, Benedix et al.^3^ found a need for a further subdivision of RCC and LCC. The study indicated that age and tumour differentiation support the common segregation into RCC and LCC, but with regard to gender, UICC stage, metastases, T- and N-status and lymphatic invasion, a subdivision into the caecum, ascending, transverse, descending and sigmoid colon is necessary. Cancers of the caecum and splenic flexure seemed more advanced (stage III/IV in the UICC classification) and more often had lymphatic invasion than cancers of the ascending and descending colon. Still, the overall picture from the present systematic literature review is that RCCs are more advanced than LCCs. The reason for this could be the weaker symptoms in patients with RCC than in patients with LCC. RCC is often associated with unnoticed bleeding, whereas LCC with changes in bowel habits, passage trouble and obstruction^6^ ^,^ ^40^. This may cause RCC patients to seek medical assistance later than LCC patients. Another factor that may delay the diagnosis in RCC is connected to colonoscopy and the documented inferior rate of success in the detection of RCC^4^. This is due to incomplete examinations in 3-13% of the patients and is thought to be responsible for half of all missed cancers^20^. The problem is most severe in older patients and especially in women^32^, which may possibly explain part of the increased prevalence of poorly differentiated cancers in advanced stages of RCC among older females.

Multivariate analyses indicated that other factors than tumour location contribute to the higher mortality in RCC^2 ,10,36,40^. These factors include age, gender, and co-morbidity, which were shown to influence the prognosis, while Benedix et al.^3^ only found little impact of location itself^2^. This was supported by Weiss et al.^40^ who found no difference in prognosis between RCC and LCC after adjustment for age, gender, co-morbidity and postoperative adjuvant chemotherapy treatment, and by Suttie et al.^36^ who found age, operative intent, mode of presentation and stage to be the only variables with a significant impact on survival in multivariate analysis.

An important limitation in this paper was the lack of molecular information in this group of patients^7^. However, molecular biological investigation have shown differences between RCC and LCC with more mutations of the C-K-RAS proto-oncogene in RCC, which, in turn, was associated with a significantly poorer prognosis, thereby indicating an impact of location itself^9^. A more recent molecular biological study found that defective DNA mismatch repair (dMMR) genes were also predominantly seen in parts of the colon located orally to the splenic flexure, which, as earlier mentioned, is a part of the embryologically derived midgut (more precisely orally from the transition between the oral two-thirds and anal one-third of the transverse colon), whereas dMMR genes were rare in the hindgut-derived descending, sigmoid colon and the rectum^15^. However, patients with dMMR had a reduced recurrence rate^4^. Microsatellite instability (MSI), has also been observed more often among RCC patients than among LCC patients^16^, and is similarly related to a better overall survival^21^ ^,^ ^28^ despite the fact that the effect of adjuvant chemotherapy, especially 5-fluouracil, is reduced in patients with MSI^38^. Further, Weiss et al.^40^ found that a lower percentage of patients with RCC than patients with LCC completed a course of adjuvant chemotherapy, probably because of their more advanced age, which, in turn, could contribute to the lower survival rate in RCC.

## CONCLUSION

This analysis on colon cancer patient’s provides evidence that the prognosis of localized left-sided colon cancer is better compared to right-sided colon cancer. Also, the patients with right colon cancer have more advanced stage, mucinous tumor and are older.
